# Effect of *Geobacillus toebii* GT-02 addition on composition transformations and microbial community during thermophilic fermentation of bean dregs

**DOI:** 10.1038/s41598-021-99413-7

**Published:** 2021-10-07

**Authors:** Xiaojia Chen, Chengjian Wu, Xiang Li, Chenyang Wang, Qinyu Li, Peng Zhou, Dong Wei, Jiping Shi, Zhijun Zhao

**Affiliations:** 1 Lab of Biorefinery, Shanghai Advanced Research Institute, Chinese Academy of Sciences, No. 99 Haike Road, Pudong, Shanghai, 201210 China; 2 School of Life Science and Technology, ShanghaiTech University, Shanghai, 201210 China; 3 University of Chinese Academy of Sciences, Beijing, 100049 China; 4 Fuzhou Kaijie Foodstuff Development Co., Ltd., Fuzhou, 350003 Fujian China; 5 Shanghai Engineering Research Center of Biotransformation of Organic Solid Waste, Shanghai, 200241 China

**Keywords:** Ecology, Microbiology, Ecology, Environmental sciences

## Abstract

Bean dregs can be prepared into organic fertilizer by microbial fermentation. *Geobacillus toebii* GT-02, which has promoting effect on bean dregs fermentation, was isolated from horse dung and it grows within a range of 40–75 °C and pH 6.50–9.50. The effectiveness of GT-02 addition on composition transformations and the microbial community in bean dregs thermophilic fermentation at 70 °C for 5 days was investigated (T1). Fermentation of bean dregs without GT-02 served as control (CK). The results showed that T1 (the germination index (GI) = 95.06%) and CK (GI = 86.42%) reached maturity (defined by GI ≥ 85%) on day 3 and day 5, respectively. In addition, the total nitrogen loss of T1 (18.46%) on day 3 was lower than that in CK (24.12%). After thermophilic fermentation, the total organic carbon and dry matter loss of T1 (53.51% and 54.16%) was higher than that in CK (41.72% and 42.82%). The mean microbial number in T1 was 4.94 × 10^7^ CFUs/g dry matter, which was 5.37 times higher than that in CK. 16S rDNA sequencing identified *Bacillus*, *Geobacillus* and *Thermobacillus* as dominant in CK, while *Bacillus*, *Ammoniibacillus* and *Geobacillus* were dominant in T1. A canonical correspondence analysis showed that *Geobacillus* and *Ammoniibacillus* were positively correlated with the GI. Thus, thermophilic fermentation with GT-02 can promote the maturity of bean dregs, which indicated the potential application value of GT-02 in thermophilic fermentation.

## Introduction

Bean, as an important oil crop and food resource, has a long history in China. Bean dregs are by-products of bean production, and 15 million tons of bean dregs are generated in China each year^1^. Bean dregs enriched with carbohydrates, protein and other nutritional components are easily decomposed and produced malodorous substances, which caused serious environmental contamination^2,3^.

Bean dregs can be used to produce active carbon, feed and organic fertilizer. To produce active carbon, bean dregs need more energy for pyrolysis^4^. Bean dregs contain trypsin inhibitors, phytic acid, tannins and other anti-nutrition factors, which restrict their use as feed additives^5^. Due to their high contents of protein, minerals and other nutrients^6^, bean dregs can be decomposed through fermentation into humus, which is considered a good fertilizer for plants^7^. Furthermore, fertilizer is green and safe due to its low pathogenicity.

Aerobic fermentation is an important method in organic solid waste compost treatment. Temperature is considered as one of the most important variables affecting aerobic fermentation efficiency^8^. High temperature can increase bioconversion efficiency, enhance organic fertilizer maturity and shorten the aerobic fermentation period and thus improve the final quality of organic fertilizer^9,10^. For example, the traditional aerobic fermentation of sewage sludge needs 24 days to achieve maturity, by compost, whereas thermophilic aerobic fermentation only needs 12 days^11^. In addition, exposure to a temperature higher than 70 °C for more than 25 min could inactivate pathogenic microorganisms in organic waste^12^. At present, there is no report about the preparation of organic fertilizer by thermophilic aerobic fermentation of bean dregs.

Microbes play a dominant role during fermentation because they have the capacity to mineralize nutrients by producing enzymes to decompose organic waste. Most microorganisms cannot live at high temperatures. Therefore, it is very important to add thermophilic bacteria in the thermophilic fermentation of bean dregs.

*Geobacillus* can be used in high-temperature treatment of organic solid waste. For example, it was reported that inoculation by *Geobacillus* strains would be effective in the thermophilic stage of vegetable waste composting^13^. In addition, inoculation with *Geobacillus stearothermophilus* would enhance the quality and efficiency of sludge fermentation^14^. *Geobacillus toebii* (*G.toebii*), which can grow on a rich complex medium at temperatures between 55 and 75 °C^15^, was initially isolated from hay compost in Korea^16^. This bacterium can produce amylase, protease, cellulase, xylanase, and mannanase, which are active at temperatures higher than 70 °C^17^. Therefore, *G. toebii* have the potential to treat organic solid waste more effectively. At present, few studies have used *G.toebii* in bean dregs fermentation to produce organic fertilizer, and the effect of *G.toebii* on the bean dregs fermentation process, particularly at a high temperature (70 °C), has not been reported.

To obtain a better understanding of *G.toebii* and improve the fermentation efficiency at high temperatures, the purposes of this study were (i) to identify the dynamics of the composition and microbial community during the thermophilic aerobic fermentation of bean dregs with *G.toebii* and (ii) to investigate the relationship between the microbial community and physicochemical indexes after adding *G.toebii*.

## Materials and methods

### Screening bean dregs degrading bacteria

Nine strains of bean dregs degrading bacteria have been obtained from different organic solid waste. Among them, two strains were from horse dung (Shanghai Wild Animal Park, Shanghai, China), three strains were from sewage sludge (Shanghai Fengxian domestic sludge treatment plant, Shanghai, China), one strain was from bean dregs (Shanghai Qingmei Food Co., Ltd., Shanghai, China), one strain was from pig manure (Shanghai Guangming Pig Farm, Shanghai, China), one strain was from dairy manure (Zhangjiagang dairy farm, Jiangsu, China), and one strain was from Chinese medicinal residue (Dhi sichuan pharmaceutical co., Jiangsu, China). Specific screening methods are as follows: the organic solid waste was inoculated in bean dregs medium (1 L water containing 10% bean dregs (v:w)) at 70 °C for 48 h. The final enriched media was diluted serially and spread on bean dregs agar plates (1 L water containing 10% bean dregs and 1.5% agar (v:w:w)). The plates were incubated at 70 °C for 12 h, and single colonies with morphological differences were selected and streaked on new plates. Different single colonies were cultured for 12 h at 70 °C and then centrifuged for 10 min at 6000 rpm to collect the corresponding bacteria. The different types of bacteria were cultured individually in 50 g bean dregs for 5 days at 70 °C. The degradation rate of dry bean dregs was investigated.

### Pylogenetic analysis

GT-02 DNA was extracted and purified by a Rapid Bacterial Genomic DNA Isolation Kit (Sangon Biotech, China). The extracted DNA was amplified (16S rDNA gene) using the primers 27F (5′-AGAGTTTGATCMTGGCTCAG-3′) and 1492R (5′-GGTTACCTTGTTACGACTT-3′). The PCR mixture contained 10.0 μL 2 × PrimeSTAR HS DNA Polymerase (Premix), 0.5 μL of each primer, 1 μL DNA template and water to 20 μL. The thermocycling conditions were as follows: 1 cycle of 5 min at 98 °C; 30 cycles of 10 s at 98 °C, 5 s at 55 °C, and 1 min 30 s at 72 °C; and a final extension step of 10 min at 72 °C. The PCR products were purified by gel electrophoresis and then sequenced by Sanger sequencing (Sangon Biotech, China). The 16S rDNA gene sequences were compared in a BLAST search to the NCBI database. Phylogenetic analysis was performed using MEGA-X software. The relationships between sequences were analysed using the neighbour-joining method. Bootstrap analysis was used to evaluate the tree topology of the neighbour-joining data by analysing 1,000 randomized data sets.

### Inoculant preparation

The strain GT-02 was activated on 50 mL CYS (pH 7.5) liquid medium^18^ and shaken (150 rpm) at 70 °C for 12 h. Then, all bacterial liquid was transferred into new 1 L CYS liquid medium and shaken (150 rpm) at 70 °C for 12 h before use. The bacteria were centrifuged for 10 min at 6000 rpm, and the supernatant was discarded. The sediments were diluted in sterile distilled water and then adjusted to OD_600_ = 1 (about 2.2 × 10^8^ colony-forming units (CFUs)/mL).

### The fermentation experiments

The fresh bean dregs used in this experiment were obtained from Shanghai Tramy Green Food Company (Pudong, Shanghai, China). The bean dregs were dehydrated to approximately 70.00% water content by a solid–liquid separator, and the pH (native pH 5.25) was adjusted to approximately 7.50 using NaOH. These bean dregs had not been sterilized. Forty millilitres (2.20 × 10^8^ CFU/mL) of inoculant was added to 1 kg of bean dregs (T1), and 40 mL of sterile water was added to another 1 kg of bean dregs as a control (CK). The physicochemical properties of the raw stocks are shown in Table 1.

**Table 1:** Physicochemical properties of the raw materials used in this study.

Parameters	CK	T1
Moisture (%)	78.51 ± 0.09	78.84 ± 0.15
pH	7.50 ± 0.23	7.52 ± 0.10
EC (μS/cm)	639.60 ± 45.46	608.95 ± 36.06
TOC (%)^a^	38.74 ± 0.48	39.43 ± 0.41
TN (%)^a^	1.76 ± 0.05	1.84 ± 0.03
C/N ratio ^a^	21.96 ± 0.44	21.43 ± 0.19
NH_4_^+^-N (mg/kg) ^a^	2207.69 ± 331.70	2289.95 ± 187.84
NO_2_^–^N (mg/kg) ^a^	712.85 ± 64.15	837.91 ± 121.80
NO_3_^–^N ^a^	–	–
Protein (%)^a^	9.64 ± 0.14	9.63 ± 0.06
Fat (%)^a^	12 ± 0.84	12.1 ± 0.07
Fibre (%)^a^	43 ± 0.12	43 ± 0.42

CK (bean dregs), T1 (bean dregs + 10% *Geobacillus toebii*), EC (electrical conductivity), TOC (total organic carbon), TN (total nitrogen), C/N ratio (carbon/nitrogen ratio), NH4 + -N (ammonium nitrogen), NO2–N (nitrite nitrogen), NO3–N (nitrate nitrogen). The data represent the means ± standard deviations from three measurements.

^a^Dry weight.

**Figure 1:**
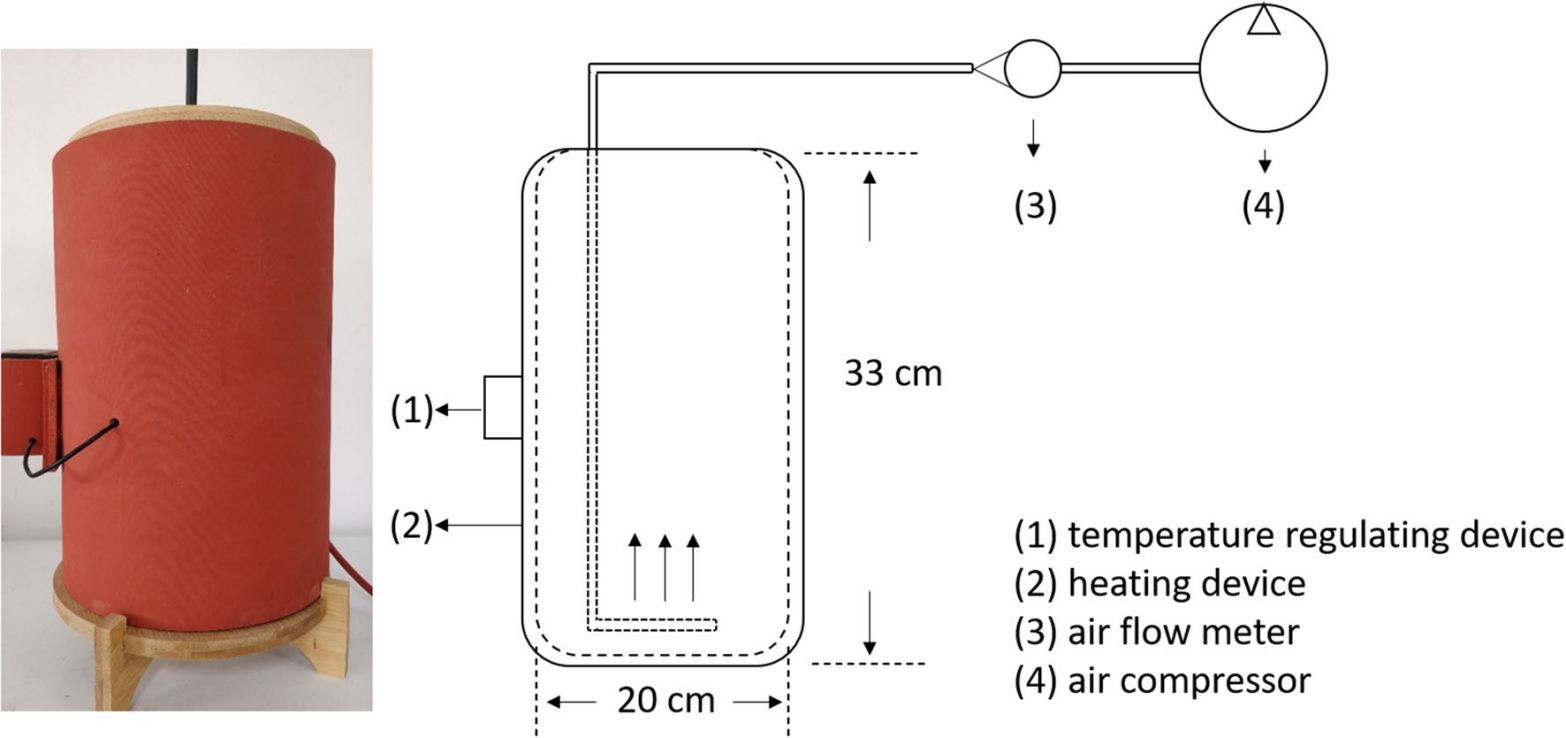
Fermentation system.

The fermentation system (Fig. 1) used in this study was designed to study the thermophilic fermentation process of bean dregs. Fermentation reactors (capacity 7.50 L, material weight 1 kg) were used in the experiment, set at 70 °C, and forced ventilation (0.50 L/min). The experimental period was 5 days. The bean dregs were poured out every 24 h, fully mixed with 100 mL sterile water to controlled approximately 75.00% moisture, and collected. Twenty grams of sample was collected on days 0, 1, 2, 3, 4 and 5 from every reactor. One part of each sample was air dried and fined through a 0.10 mm sieve for physicochemical parameter determination, and the other part was used for microbiological analysis. Each treatment was performed in triplicates.

### Physicochemical properties analysis

The weight of each pile was recorded using an electronic balance, and the dry matter weight was then calculated based on the pile weight and moisture content. The moisture content of samples was determined after drying at 105 °C for 24 h by the drying-weighing method^19^. According to the dry weight content of bean dregs, the moisture content of samples was adjusted to about 75.00% by adding sterile water, and then per gram sample was mixed with 10 mL of sterile water, and the mixture was oscillated for 10 min. One hundred microlitres of the liquid mixture was diluted and cultivated on CYS liquid medium at 70 °C for 24 h to observe the number of colonies on the plate. Another liquid mixture was centrifuged for 10 min at 6000 rpm, and the supernatant was used to determine pH, electrical conductivity (EC) and the germination index (GI). The germination test was run using seeds of carrot, and the GI was calculated as described by Li et al.^20^. The suspensions were filtered through a 0.45 μm membrane, and the filtrates were used for characterization of dissolved organic matter (DOM) by a fluorescence spectrometer (F-7000, Hitachi). Spectra were recorded at a scan rate of 2400 nm/min using Ex and emission (Em) slits. Wavelengths were set from 200 to 450 nm for Ex and from 250 to 550 nm for Em. A photomultiplier tube voltage of 400 V was applied for low-level light detection. The Fourier transform infrared (FTIR) spectra of dry matter were measured from 4000 to 600 cm^−1^ at 2 cm^−1^ by a Spectrometer II (PerkinElmer, USA). Total nitrogen (TN) and total organic carbon (TOC) were determined as described by Yu et al.^21^. The protein content was estimated by multiplying the content of organic nitrogen (TN minus NH_4_^+^-N) by 6.25^22^. The ammonium (NH_4_^+^-N), nitrite nitrogen (NO_2_^–^N), nitrate nitrogen (NO_3_^–^N), fat content and crude fibre content were measured using the standard methods established by the International Standardization Organization^23–25^. The loss rate formula of TN, protein fat and fibre is calculated as the follow:

$$\mathrm{The loss rate }\left(\mathrm{\%}\right)=\left[1-\frac{{m}_{5d}\times \left(1-{c}_{5d}\right)}{{m}_{0d}\times \left(1-{c}_{0d}\right)}\right]\times 100$$

m_0d_ (units: g) and m_5d_ (units: g) represent the initial mass of bean dregs and the mass after fermentation for 5 days, respectively. c_0d_ and c_5d_ represent the initial TN, protein, fat or fibre content of bean dregs and the content after fermentation for 5 days, respectively. All masses are calculated by wet weight.

### Microbial community analysis

The three replicates of fermentation samples were mixed evenly according to the same dry matter mass. The fermentation samples were subjected to DNA extraction, 16S rDNA gene amplification, and subsequent microbial community analysis by Illumina sequencing. Total genomic DNA was extracted from 0.5 g fermentation samples using the cetyltrimethylammonium bromide (CTAB) method (E.Z.N.A.^®^ soil DNA Kit, Omega Bio-Tek, Norcross, GA, USA) and the extracted DNA was used as the template for polymerase chain reaction (PCR). The PCR mixture contained 4 μL of 5 × FastPfu Buffer, 2 μL of 2.5 mM dNTPs, 0.8 μL of each primer (5 μM), 0.4 μL of FastPfu Polymerase, 10 ng of template DNA and water to 20 μL. The V4-V5 region of the 16S rDNA gene was amplified using the primers 515F (5′-GTGCCAGCMGCCGCGGTAA-3′) and 907R (5′-CCGTCAATTCMTTTRAGTTT-3′). The PCR conditions were as follows: 1 cycle of 3 min at 95 °C; 27 cycles of 30 s at 95 °C, 30 s at 55 °C, and 45 s at 72 °C; and a final extension step of 10 min at 72 °C. The PCR products were extracted by electrophoresis on 2% agarose gel and purified with an AxyPrep DNA Gel Extraction Kit (Axygen Biosciences, Union City, CA, USA). To calculate the appropriate sample volume for sequencing, the resulting DNA products were quantified using Quantus™ Fluorometer (Promega, USA).

According to the standard protocols in Majorbio Bio-Pharm Technology Co. Ltd. (Shanghai, China), purified PCR products were analyzed on an Illumina MiSeq platform (PE300, Shanghai Personal Biotechnology Co., Ltd.) for microflora analysis. Raw Illumina fastq files were demultiplexed, quality-filtered, and analyzed using Quantitative Insights Into Microbial Ecology analysis (version 1.9.1 http://qiime.org/). Subsequently, to remove chimera and determine operational taxonomic units (OTUs) concurrently, the quality-filtered sequences were clustered into OTUs with a 97% similarity cut-off using UPARSE (version 7.1 http://drive5.com/uparse/). The 16S rDNA sequences were analysed and annotated by RDP Classifier (version 2.2 http://sourceforge.net/projects/rdp-classifier/) against the SILVA (SSU138) 16S rDNA database with a confidence threshold of 70%^10^.

### Statistical and bioinformatic analysis

SPSS v.19.0 was used for calculating the significance tests of differences of physicochemical indexes using a one-way analysis of variance (ANOVA). Significant differences between each treatment were determined by the Tukey test (α = 0.05 and *P* < 0.05). Origin 2021 was used to prepare the figures. Canonical correspondence analysis (CCA) was performed using the Canoco 5.0 software package.

## Results and discussion

### Isolation and characterization of bean dreg-degrading strains

**Figure 2:**
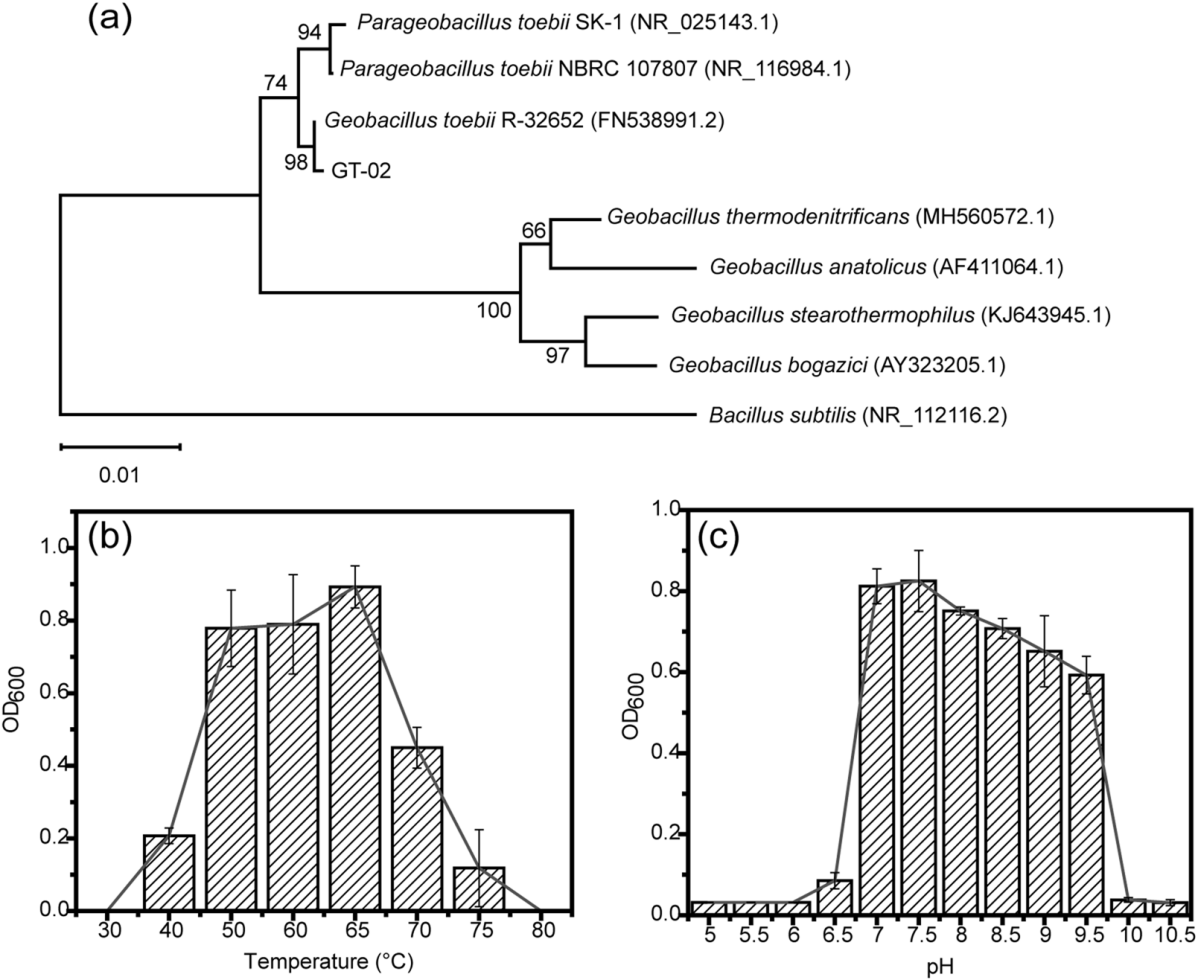
**(a)** Phylogenetic tree based on 16S rDNA gene sequences from related species of the genus *Geobacillus* constructed using the neighbour-joining method with 1000 bootstrap replicates. Branch length is indicated at each node. **(b)** The growth curve of strain GT-02 with temperature. **(c)** The growth curve of strain GT-02 with pH.

A 1362-bp amplification fragment of 16S rDNA was obtained by PCR (GenBank accession number MW406939). This sequence was compared with others in the GenBank database, aligning the 16S rDNA sequences with several *Geobacillus sp.* strains and constructed a phylogenetic tree (Fig. 2a). The phylogenetic tree clearly showed that strain GT-02 belongs to the *G.toebii* branch and was similar to *G.toebii* R-32652, *G.toebii* NBRC 107807, and *G.toebii* SK-1 with 99.78%, 99.63% and 99.05% similarities, respectively. According to the study described previously, *G.toebii* was a gram-positive, aerobic rod and motile bacterial^26^. *G.toebii* could produce acid from inositol and gas from nitrate. *G.toebii* could hydrolysis casein and utilize n-alkanes as carbon source^27^.

The growth characteristics of strain GT-02, such as temperature and pH values, were investigated. The bacterial strain could grow within a range of 40–75 °C and pH 6.50–9.50, and the optimum temperature and pH were 65 °C and 7.50, respectively (Fig. 2b,c). Compared to other *G.toebii* strains, the maximum growth temperature and pH of strains R-32652 and SK-1 were 70 °C and 9.00^26,28^, respectively. These results showed that strain GT-02 was more resistant to high temperature and alkalinity. Fermentation temperature above 70 °C could effectively inactivate harmful microorganisms in organic solid waste^12^. Therefore, the fermentation temperature was set at 70 °C in this study.

### Changes in the composition of bean dregs during fermentation

#### Changes in GI, TOC and TN of bean dregs during fermentation

**Figure 3:**
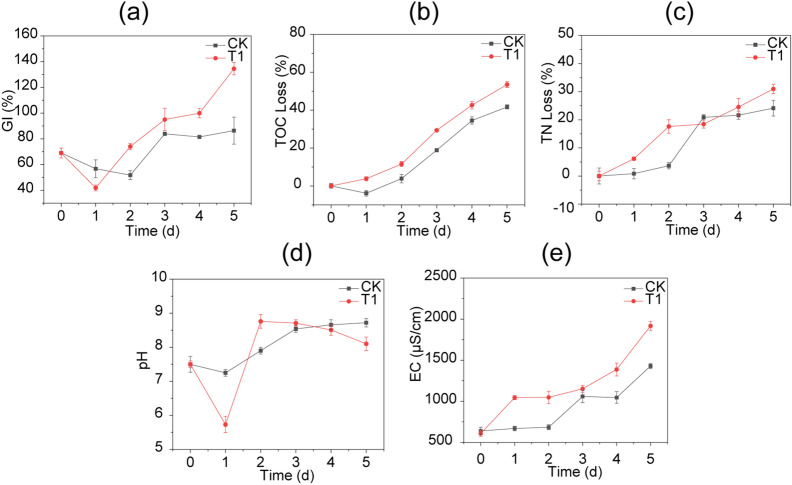
Profiles of GI **(a)**, TOC **(b)**, TN **(c)**, pH **(d)** and EC **(e)** during the fermentation process of CK and T1. The data represent the means ± standard deviations from three measurements.

The GI is traditionally used to evaluate the phytotoxicity and maturity of organic fertilizer^12^. As shown in Fig. 3a, both groups of experiments reached the standard of maturity (GI ≥ 85.00%). Therefore, the fermentation was terminated in five days. In the initial stage of fermentation, the GI of CK dropped to 51.85% on day 2, and the GI of T1 dropped to 41.98% on day 1. Phytotoxicity, which is usually caused by various heavy metals and low-molecular-weight substances, such as NH_3_ and organic acids, can reduce seed germination and inhibit root development^29^. During fermentation, bean dregs might produce NH_3_, organic acids and other substances, which could trigger a decrease in the GI. The GI of T1 showed a clear decrease, which was likely due to the production of toxic organic acids and might also explain the decrease in pH observed in T1 (Fig. 3d). Due to the degradation of organic acids, the GI of T1 increased to 95.06% on the third day and continued to increase to more than 100.00%, whereas in CK, the GI only reached 86.42% at the end of the fermentation. These results revealed that the maturity of T1 on day 3 was markedly higher than that of CK on day 5 and thus suggest that *G.toebii* can significantly enhance the fermentation efficiency by accelerating the maturation process and thus reducing the thermophilic fermentation period from 5 to 3 days.

TOC is usually used as an energy source by microorganisms^30^. The TOC loss in both CK and T1 increased during fermentation (Fig. 3b). The reduction of TOC was mainly caused by the production of carbon dioxide from bacterial respiration. The rate of TOC loss in T1 was higher than that in CK. At the end of the fermentation, the TOC loss of T1 was 11.78% higher than that in CK. Because of the addition of *G.toebii*, bacterial metabolism in T1 was more active, and organic degradation was faster.

The TN loss in both CK and T1 also showed an upward trend (Fig. 3c). The loss of TN was mainly caused by the volatilization of ammonia nitrogen^31^. The rate of TN loss in T1 increased more than that of CK group. After fermentation (day 5), the TN loss in T1 was 6.83% higher than that of CK. The mineralization in T1 was more active and thus ammonia nitrogen was more, which was easy to cause volatilization. However, the bean dregs in CK were mature on the 5th day, while those in T1 were on the 3rd day. At this time, the TN loss of mature bean dregs in T1 was 5.66% lower than that in CK, which indicated that the bean dregs lost less nitrogen source when they reached the standard of maturity after the addition of *G.toebii*.

#### Changes in pH and EC of bean dregs during fermentation

The variation in pH observed during fermentation is due to the interaction between inorganic nitrogen and organic acids produced by the decomposition of organic matter^32^. As shown in Fig. 3d, the pH of CK gradually increased to 8.72 at the end of the fermentation. The ammonification process and the release of free NH_3_ during organic matter (OM) degradation lead to increases in pH^33^. The pH of T1 decreased to 5.73 on day 1, which was due to the formation of more organic acids than CK, and then increased to 8.76 on day 2, which was due to acid consumption and ammonia formation. Figure 2c showed that GT-02 could hardly grow when the pH was lower than 6.00, but the heterogeneity of solid fermentation provided a possible living environment for the growth of GT-02. Subsequently, the pH of T1 slowly decreased to 8.10 due to ammonia volatilization or ammonia conversion. These study findings showed that the pH value of the fermentation process was significantly affected by the addition of GT-02. *G.toebii* can produce abundant high-temperature enzymes, such as amylase, protease, cellulase, xylanase, and mannanase^17^, which explains why the ammonification process was faster in T1 than in CK and thus the higher pH was found in T1.

The EC, which is a measure of the total ion concentration, describes changes in the levels of organic and inorganic ions such as SO_4_^2−^, Na^+^, NH_4_^+^, K^+^, Cl^−^, and NO^3−^ during the fermentation process^34^. As shown in Fig. 3e, the EC of the two groups increased significantly during fermentation process (*P* < 0.05). The increase in EC observed in this study was due to the decomposition of a fraction of OM into mineral salts and ammonium ions, and these results showed that thermophilic fermentation can effectively promote the degradation of bean dregs. Bean dregs inoculated with GT-02 exhibit enhanced degradation. At the end of the fermentation, the EC of T1 was higher than that of CK (*P* < 0.05), and the higher ion concentration of T1 could provide more nutritional factors for plant growth.

### Nitrogen transformation process of bean dregs during fermentation

**Figure 4:**
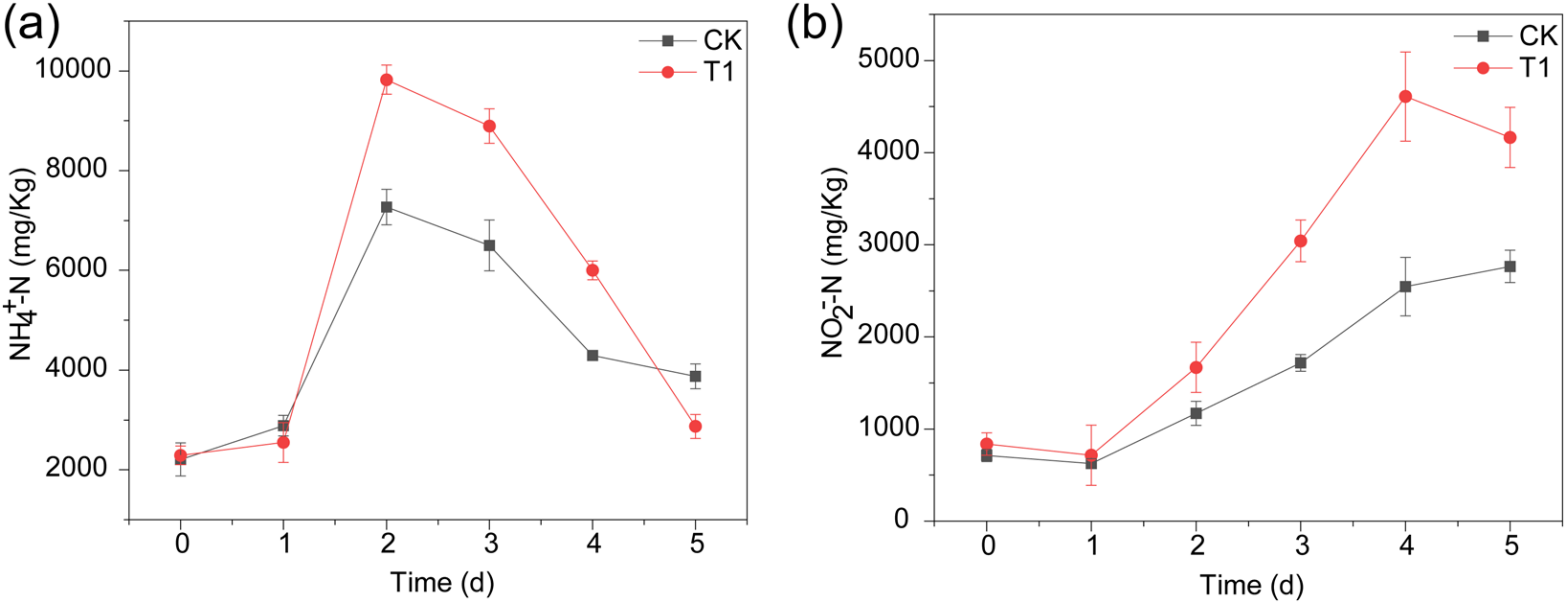
Profiles of NH_4_^+^-N **(a)** and NO_2_^–^N **(b)** during the fermentation process of CK and T1. The data represent the means ± standard deviations from three measurements.

The TN in fermentation samples generally includes organic nitrogen, ammonia nitrogen (NH_4_^+^-N), nitrite nitrogen (NO_2_^–^N) and nitrate nitrogen (NO_3_^–^N). As shown in Fig. 4a, a rapid increase in the NH_4_^+^-N concentration was observed during the initial fermentation process until a peak was reached after 2 days in CK (*P* < 0.05), which could be ascribed to the mineralization of organic nitrogen in bean dregs and weak ammonia oxidation at high temperature^35,36^. The increase in the NH_4_^+^-N content detected in T1 during the first 2 days was faster than that in CK. A possible reason for this finding was that GT-02 rapidly decomposed the nitrogenous organic compounds to generate a large amount of NH_4_^+^-N. The NH_4_^+^-N content in both CK and T1 decreased rapidly from days 2 to 5 in CK (*P* < 0.05), which was possibly due to ammonia oxidation to NO_2_^–^N and the volatilization of NH_3_.

As shown in Fig. 4b, the NO_2_^–^N content in CK continued to increase during fermentation, which was likely due to the ammonia oxidation activity of thermophilic bacteria. The NO_2_^–^N concentration of T1 rapidly increased to 4608.42 mg/kg on the fourth day. It’s reported that NH_4_^+^-N could be oxidized into hydroxylamine through ammonia monooxygenase^37^, and then further oxidized into NO_2_^–^N by hydroxylamine oxidase^38^. It is suspected that the addition of GT-02 stimulated the expression of ammonia monooxygenase and hydroxylamine oxidase and thus accelerated the transformation of NH_4_^+^-N to NO_2_^–^N. Subsequently, the NO_2_^–^N concentration of T1 decreased from 4608.42 to 4165.31 mg/kg at the end of the fermentation process, and this decrease can be mainly attributed to the transformation of NO_2_^–^N to other substances.

NO_3_^–^N was not detected during fermentation because nitrite oxidation was limited by the high temperatures and a lack of nitrifying bacteria. Yang et al. clearly confirmed negative correlations between NO_3_^–^N and both the temperature and pH^7^. In this high-temperature and alkaline environment, which restricts the activity of nitrifying bacteria, NO_3_^–^N is not easily generated.

### Changes in the organic composition of bean dregs during fermentation

**Figure 5:**
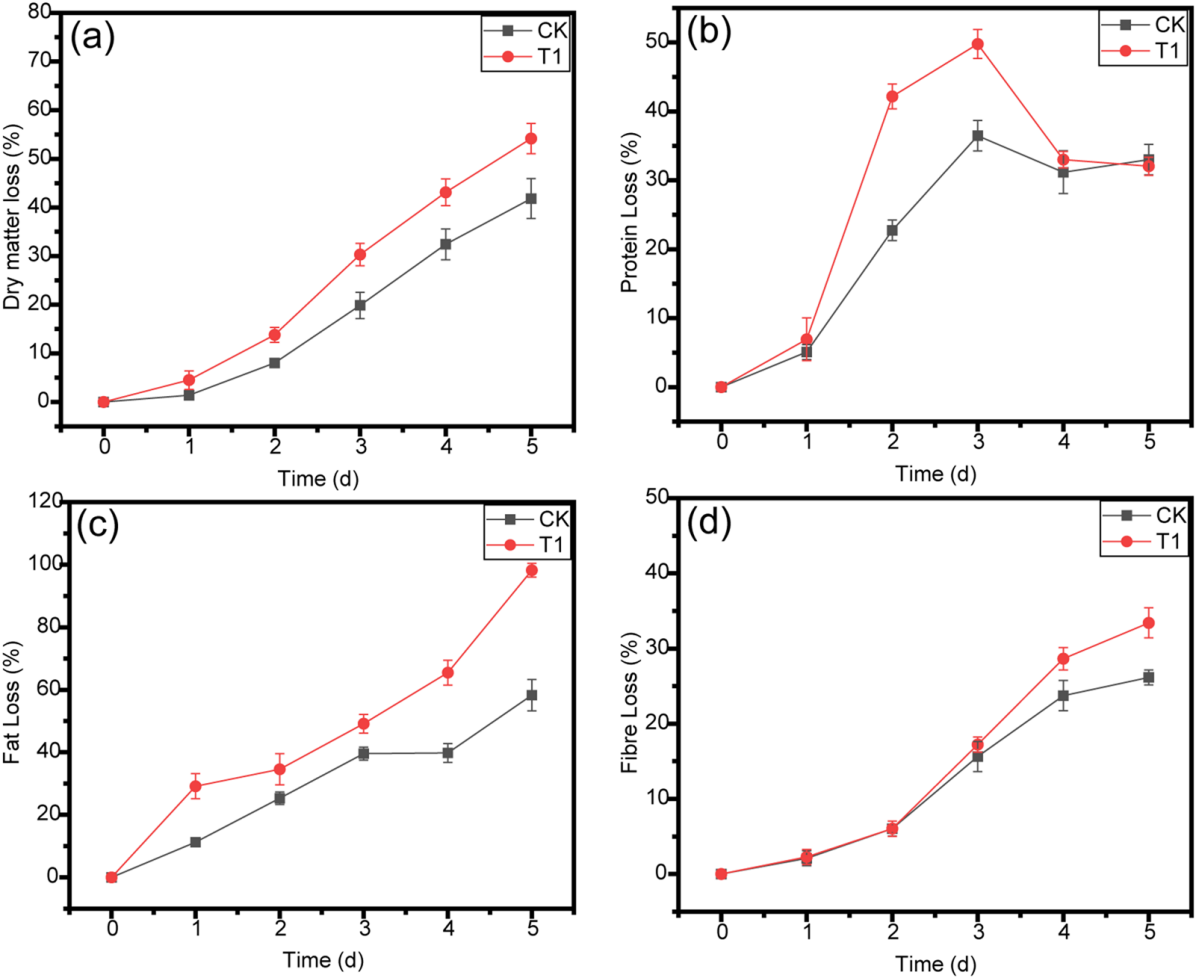
Profiles of dry matter loss **(a)**, protein loss **(b)**, fat loss **(c)** and fibre loss **(d)** in CK and T1 during fermentation process. The data represent the means ± standard deviations from three measurements.

Dry matter loss is an important parameter reflecting the degradation of bean dregs. As shown in Fig. 5a, the dry matter loss showed similar trends during the degradation of bean dregs in both T1 and CK. Dry bean dregs were degraded by 42.82% after 5 days at 70 °C without the addition of exogenous thermophilic bacteria. This meant that fresh bean dregs contained a few thermophilic microbes. In T1, the dry matter loss was 54.16% at the end of the fermentation. The degradation rate of dry matter in T1 was 11.33% higher than that in CK. These results revealed that GT-02 could grow on bean dregs and significantly contributed to the degradation of OM.

The dry weight changes were influenced by the organic composition, including protein, fat and fibre. The protein, fat and fibre losses in both experiments are shown in Fig. 5. As shown in Table 1, the dry matter of bean dregs contained 9.64% protein, which could provide nutrition for microbes. As illustrated in Fig. 5b, the protein loss in CK gradually increased (*P* < 0.05), which indicated that the growth of microbes consumed the protein in bean dregs. T1 contained more microbes than CK, and the protein loss in T1 exceeded that in CK on the first day. On the third day, the protein loss in T1 reached the maximal value (49.78%), whereas the value in CK was only 36.47%. This result indicated that the available protein in T1 was basically completely consumed by the third day. After 3 days, the protein loss in T1 decreased (*P* < 0.05). The possible reason was that more enzyme proteins were synthesized by microorganisms.

The fat loss in both CK and T1 increased during fermentation (Fig. 5c). The fat loss in T1 clearly increased faster than that in CK. Moreover, the fat in T1 was basically consumed completely at the end of the fermentation process, whereas the fat loss in CK was only 58.28%. These results indicated that GT-02 could secrete high-temperature-resistant enzymes and thereby accelerate the degradation of fat.

During the initial period of fermentation process, the fibre loss in T1 was basically the same as that in CK, as shown in Fig. 5d. A possible reason for this finding was that fibre was difficult to degrade. Starting on the third day, the difference in fibre loss between T1 and CK was more obvious. Notably, the fibre loss in T1 was 7.26% higher than that in CK at the end of the fermentation process. The increased fibre loss in T1 illustrated that more cellulases were synthesized and secreted. These enzymes are likely associated with proteins, which might explain the increase in the protein content in T1 starting on the fourth day.

The above-described results indicated that GT-02 tends to utilize easily degradable OM, such as proteins and fats, and it has the ability to degrade fibres. Therefore, during the thermophilic fermentation of bean dregs, the addition of GT-02 could accelerate the degradation of OM.

### Spectral analysis of the composition during bean dregs fermentation

#### Analysis of the effects of *G. toebii* on the DOM of bean dregs by EEM fluorescence spectra

**Figure 6:**
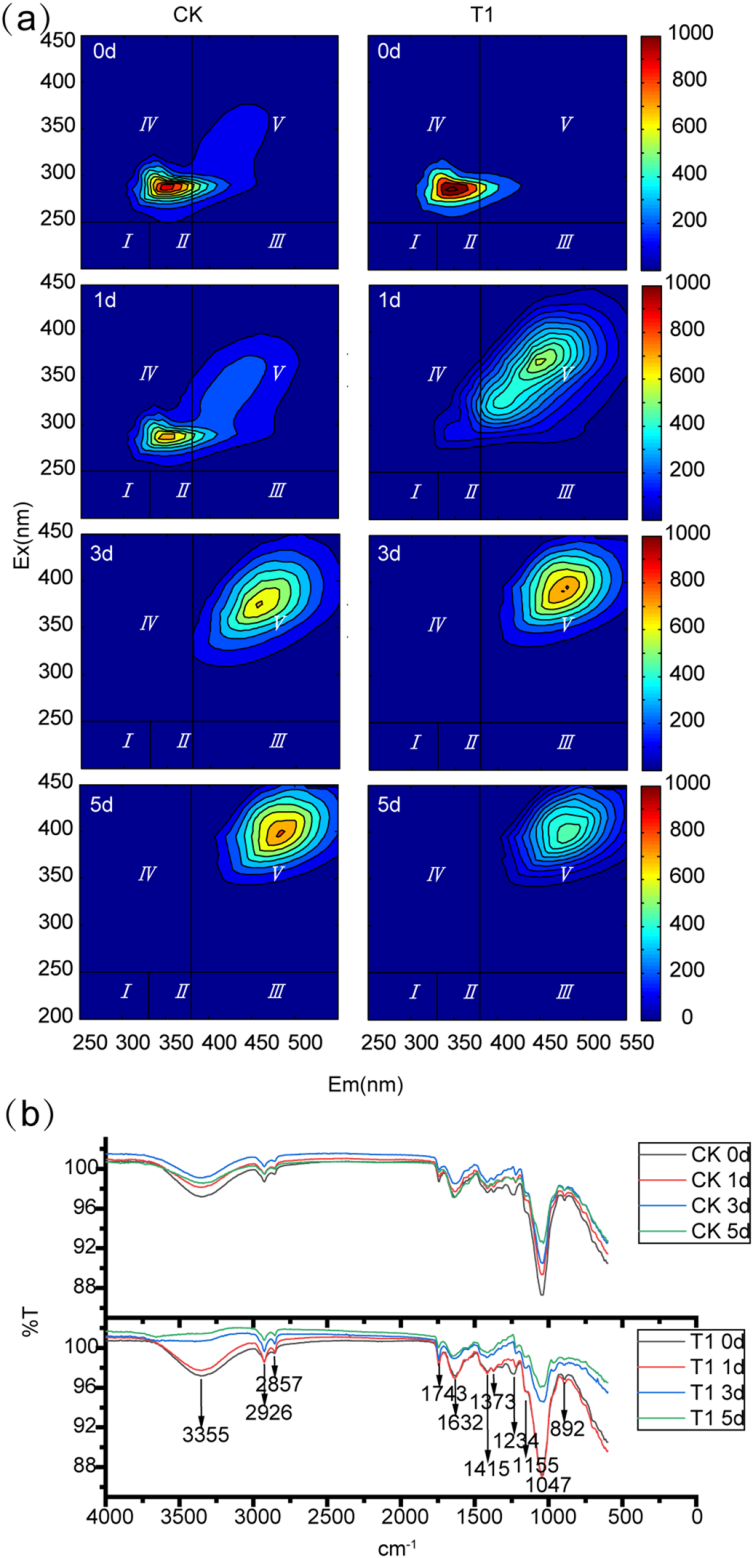
**(a)** Fluorescence excitation-emission matrix spectra of dissolved organic matter in CK and T1. Em (nm): emission wavelength (nm); Ex (nm): excitation wavelength (nm). Regions I and II: simple aromatic proteins; Region III: fulvic acid-like substances; Region IV: soluble microbial byproducts; and Region V: humic acid-like substances. **(b)** The 4000-to-600-cm^−1^ regions of the Fourier transform infrared spectra of CK and T1.

According to the study conducted by^39^, the EEM fluorescence spectrum can be delineated into five Ex/Em regions. The fluorescence peaks that fall in Regions I and II (Ex < 250 nm, Em < 380 nm) are associated with simple aromatic proteins such as tyrosine, whereas peaks that fall in Regions III (Ex < 250 nm, Em > 380 nm), IV (250 < Ex < 280 nm, Em < 380 nm) and V (Ex > 250 nm, Em > 380 nm) are related to fulvic acid-like substances, soluble microbial by-products and humic acid-like substances, respectively. The EEM spectrum of DOM from bean dregs is shown in Fig. 6a. At the beginning of fermentation, the similar EEM profiles of CK and T1 indicated that the DOM of bean dregs consisted of soluble microbial by-products and small amounts of humus substances. Moreover, the fluorescence intensity of T1 was higher than that of CK due to the addition of GT-02 (*P* < 0.05). As the fermentation process proceeded, the peak of CK in Region I became weaker until it disappeared on day 3, whereas a peak was detected in Region V. This finding indicated that during the thermophilic fermentation process, soluble microbial by-products are degraded or used for the synthesis of humic acid-like substances, which is similar to the results obtained by Yu et al.^11^. As the fluorescence peak of humic acids increased, the corresponding peak fluorescence intensity of soluble microbial by-products gradually disappeared. According to the study conducted by Jouraiphy et al.^40^, the peak in Region IV disappeared, reflecting a degradation of labile OM, and microbial activity is favoured by high concentrations of labile OM. Therefore, microbial activity would cause the disappearance of the peak in Region IV as the fermentation process proceeds.

However, for T1, the fluorescence peak in Region IV disappeared on the first day, and a new peak appeared in Region V. This finding might be due to the addition of *G.toebii*, an exogenous bacterium that significantly degrades soluble DOM in microorganisms or uses it to synthesize humic acids. Moreover, on the third day, the fluorescence intensity of Region V obtained for T1 reached the maximal value, and this value was higher than that found for CK (*P* < 0.05). This finding indicated that *G.toebii* can promote the synthesis of humic acids and improve the maturity of fermentation products. Subsequently, at the end of the fermentation process, the fluorescence intensity in Region V obtained for T1 showed a slight decrease, which might be because the produced humic acids decompose easily at high temperatures. The thermophilic fermentation process should be terminated as soon as possible after the products reach maturity. *G.toebii* significantly accelerated the degradation of labile OM and then promoted the formation of humus substances, which suggests that the humification process in T1 was faster than that in CK.

#### Analysis of the effects of *G. toebii* on the composition of bean dregs by FTIR spectroscopy

The FTIR spectra in the 4000-to-600-cm^-1^ region were evaluated in this study. Figure 6b showed the FTIR bands of the bean dregs in the CK and T1 groups during the fermentation process. The FTIR spectra of the bean dregs exhibited nine predominant absorption peaks at 3355, 2926, 2857, 1743, 1632, 1415, 1373, 1234 and 1047 cm^−1^ and two small peaks at 1155 and 892 cm^−1^. The FTIR spectra depict a broad band centred at 3355 cm^−1^ corresponding to the O–H stretching vibration, whereas the peak at 1743 cm^−1^ was attributed to strong C=O stretching of carboxylic acids or ketones^41^. The band at 1632 cm^−1^ was generated by the asymmetric COO^−^ stretching of carboxylic acids, whereas the peak at 1415 cm^−1^ was generated by the symmetric COO^−^ stretching of deprotonated carboxylic acid^42^. The peak at 1234 cm^−1^ was attributed to C-O stretching and O–H deformation of carboxyl groups and C–O stretching of aryl ethers^11^. These peaks suggested that organic acids existed in the raw bean dregs materials. Compared with the results obtained for CK, the disappearance of the peak at 3350 cm^−1^ and the greater decreases in the peaks at 1715, 1632, 1415 and 1234 cm^−1^ observed in the T1 spectra illustrated that organic acids were significantly decomposed during fermentation process (*P* < 0.05). Above all, these results indicated that the addition of GT-02 accelerated the degradation of organic acids.

The change at 1373 cm^−1^ could be attributed to the C-N stretching of amines^43,44^, and the peak at 892 cm^−1^ was caused by the strong C-H stretching of aromatics and the strong and broad N–H stretching of amines I and II^45^. The peaks at 1373 and 892 cm^−1^ decreased in the T1 spectra during fermentation (*P* < 0.05) but remained basically unchanged in the CK spectra. These peaks suggested that crude proteins were rapidly degraded by *G.toebii*. Another peak at 1155 cm^−1^, which was obviously decreased in T1 (*P* < 0.05), was attributed to the C-N stretch of aliphatic amines^45^. This result showed that aliphatic amines might be partly transformed into ammonia nitrogen by *G.toebii*. Additionally, the peaks at 2926 and 2857 cm^−1^ were attributed to symmetric and asymmetric stretching vibrations of aliphatic C-H bonds in CH_3_ and CH_2_ groups, whereas the peak at 1047 cm^−1^ was attributed to C–O stretching of polysaccharides^11,41^. These peaks gradually decreased (*P* < 0.05), which indicated that microbes consumed part of the polysaccharides and fats in both CK and T1 during the fermentation process. Moreover, the peaks in the T1 spectra decreased faster than those in the CK spectra during fermentation process, which demonstrated that the addition of *G.toebii* accelerated the degradation of polysaccharides and fats. Polysaccharides, their degradation products and nitrogenous compounds are the main precursors for the formation of humus substances^46^. Therefore, protein and polysaccharides might constitute the key structures that lead to the acceleration of the formation of humus substances in T1, which could explain the higher GI obtained with T1 compared with CK.

### Change in the bacterial community during bean dregs fermentation

**Figure 7:**
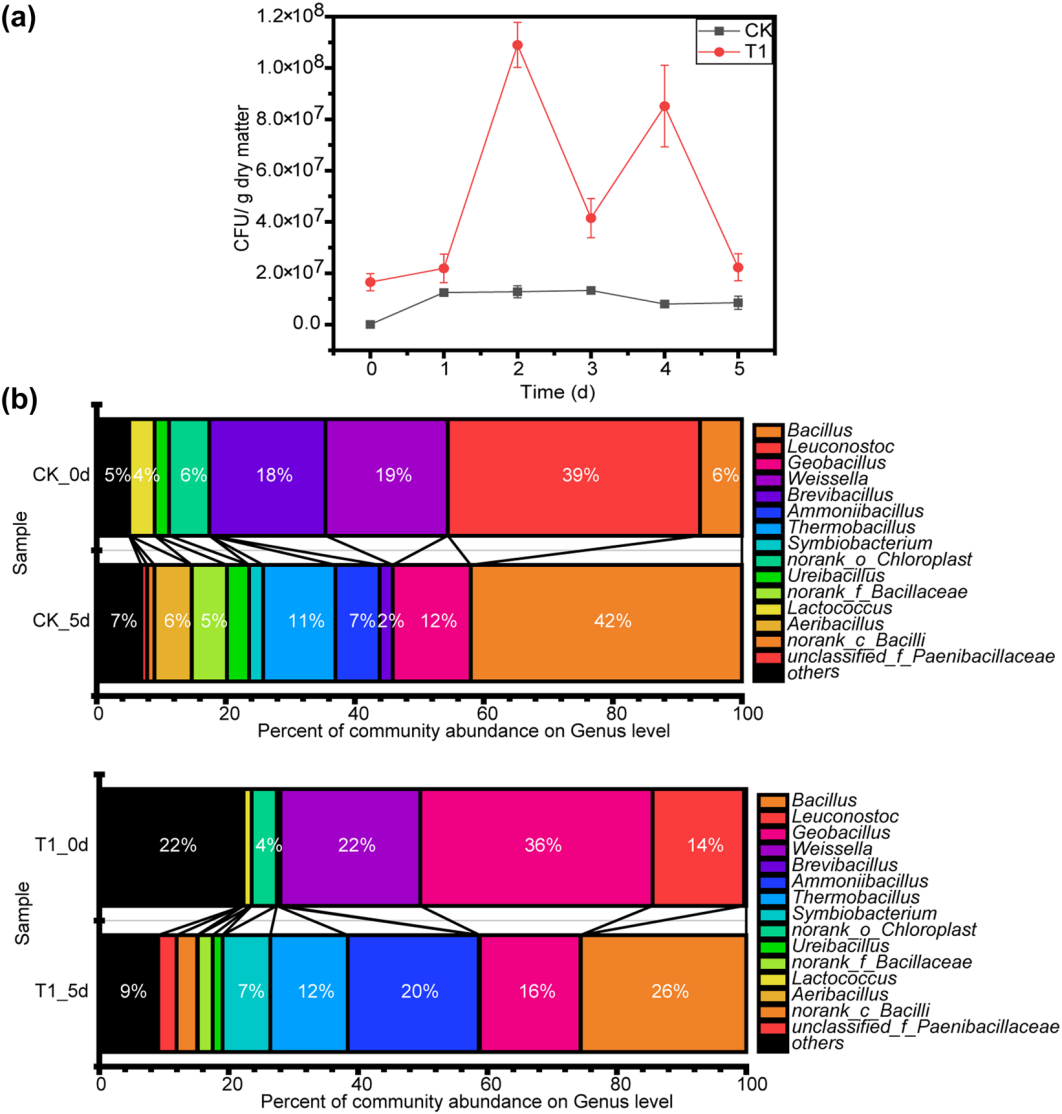
**(a)** Profiles of CFUs in CK and T1 during fermentation process. **(b)** Taxonomic classification at the genus level of predominant 16S rDNA gene sequences (relative abundance, top 15) in CK and T1. The data represent the means ± standard deviations from three measurements.

Microbes play a dominant role in the degradation of dry matter during fermentation^47^, and CFUs are used to count living bacteria. There were some thermophilic bacteria in fresh bean dregs. As shown in Fig. 7a, the concentration of bacteria in CK increased from 4.75 × 10^4^ to 1.33 × 10^7^ CFUs/g dry matter during the first 3 days of fermentation process and then decreased to reach a value of 8.53 × 10^6^ CFU/g at the end of the fermentation process. According to Poli et al.^15^, *G.toebii* can grow at a high temperature between 55 and 75 °C, which meant it could coexist with endogenous thermophilic bacteria in bean dregs at 70 °C. In T1, the number of living microbes was initially 1.65 × 10^7^ CFUs/g dry matter, which was nearly 348-fold higher than that in CK, reached the maximal value (1.09 × 10^8^ CFUs/g dry matter) on the second day, which was eightfold higher than that in CK and then declined over time until the end of the fermentation process. During the 5-day fermentation process, the mean number of living microbes in T1 was 4.94 × 10^7^ CFUs/g dry matter, which was 5.37 times that in CK due to the addition of GT-02. Therefore, due to a high concentration of thermophilic bacteria, the degradation of bean dregs in T1 was faster than that in CK.

The V4-V5 regions of the 16S rDNA gene were determined by Illumina MiSeq sequencing to characterize the microbial community structure during fermentation process. The genus-level phylogenetic characteristics of CK and T1 are compared in Fig. 7b, and the figure displays the relative abundances of various microbes in the communities during thermophilic fermentation process. The composition of the bacterial communities of CK and T1 showed obvious differences (*P* < 0.05). Prior to fermentation process, *Leuconostoc* (39%), *Brevibacillus* (18%) and *Weissella* (19%) were the dominant genera in bean dregs. At the end of the fermentation process, the microbial community in CK was significantly changed. Specifically, the percentages of *Leuconostoc*, *Brevibacillus* and *Weissella* markedly decreased to 0.03%, 2.03% and 0.01%, respectively, after five days of fermentation, and these populations were replaced by *Bacillus* (42%), *Thermobacillus* (11%), *Geobacillus* (12%) and *Ammoniibacillus* (7%). However, the original content of *Bacillus* in bean dregs was 7%, and the initial abundances of *Thermobacillus*, *Geobacillus* and *Ammoniibacillus* were basically 0%. Previous studies have shown that these four genera contain many thermophilic bacteria, such as *Bacillus* sp*. PPS-52*, *Thermobacillus xylanilyticus*, *Ammoniibacillus agariperforans* and *G.toebii*^48,49^. Thermophilic fermentation significantly increased the proportion of these bacteria (*P* < 0.05), which indicated that these four genera played an important role in the degradation of bean dregs. *Acinetobacter* (0.04%) was found in fresh bean dregs, which could cause various infections and survive exposure to various common disinfectants^50^. After thermophilic fermentation process, this species was not detected in CK and T1, which indicated that a high temperature could kill pathogenic bacteria in bean dregs.

GT-02 was added to T1 at the beginning of the fermentation process, and *Geobacillus* increased in T1 at the end of the fermentation process, which indicated that *G.toebii* exhibits good compatibility with bean dregs. After thermophilic fermentation, the microbial community structure of T1 was similar to that of CK, and the main genera were *Bacillus* (26%), *Ammoniibacillus* (20%), *Geobacillus* (16%) and *Thermobacillus* (12%), but the abundances were changed. During the thermophilic fermentation of bean dregs, the abundances of microorganisms belonging to *Geobacillus* remained high, which may explain why the rate of composition transformation in T1 was faster than that in CK. At the end of the fermentation process, the proportion of *Ammoniibacillus* in T1 was markedly higher than that in CK (*P* < 0.05). *Ammoniibacillus *gen. nov. and *Ammoniibacillus agariperforans* sp*. *nov. can utilize ammonium but not nitrate, nitrite, urea or glutamate for growth^48^, which might explain why the T1 group showed a faster decrease in the ammonia nitrogen content than the CK group. Therefore, the effects of *G. toebii* on the microbial community structure of bean dregs were significant and might both improve the growth of *Ammoniibacillus* and promote the nitrogen transformation process.

### Canonical correlation analysis of the relationship between the microbial community and bean dregs composition

**Figure 8:**
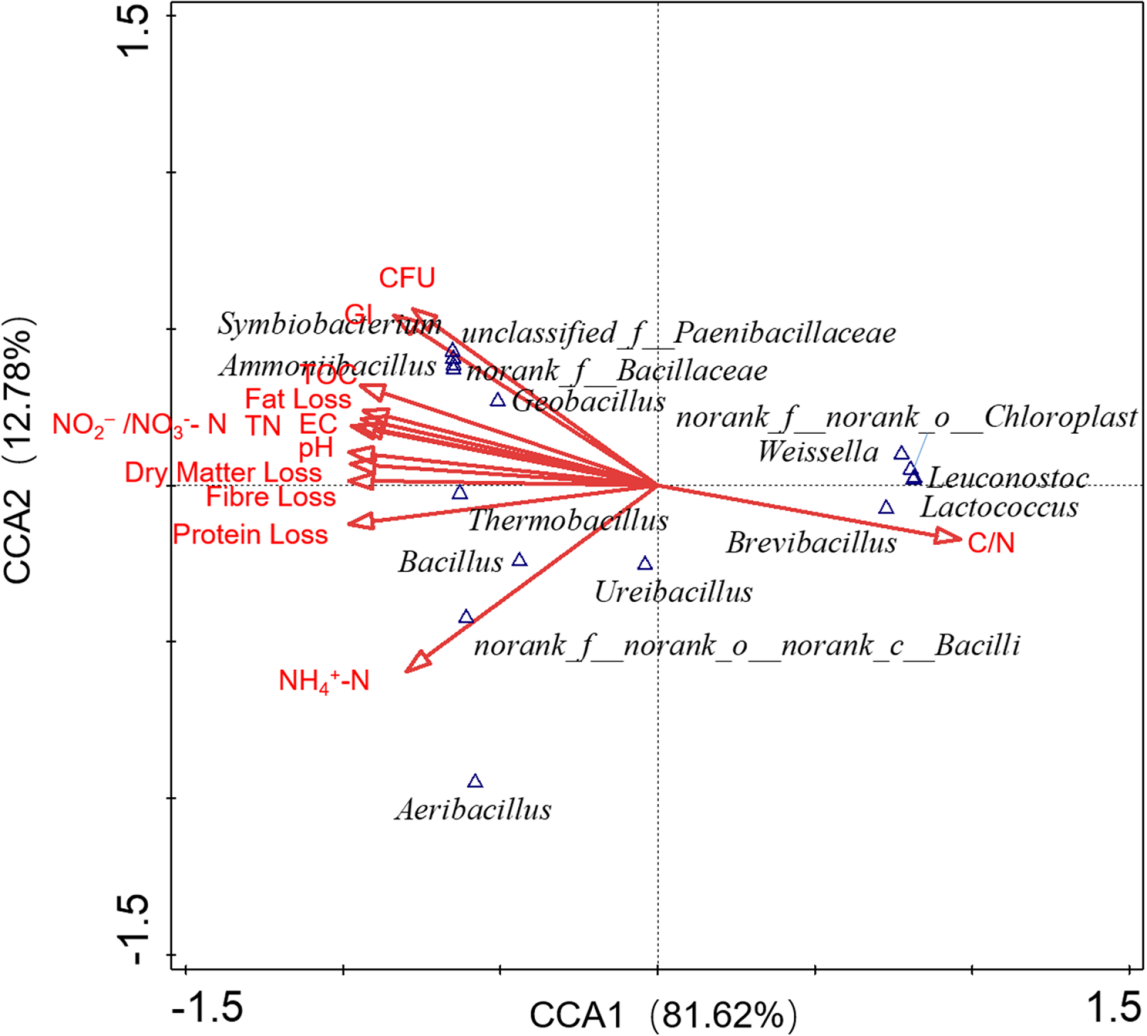
CCA between the composition and microbial community of CK and T1.

A CCA can identify the relationships among environmental factors and the microbial community. The first two axes in the CCA plot shown in Fig. 8 explain 94.40% of the cumulative variance between the species data and environmental variables. The results indicated that the GI was significantly correlated with the CFUs. The CFUs effectively promoted the maturity of bean dregs during thermophilic fermentation; thus, the maturity of T1 was higher than that of CK. *Geobacillus*, *Ammoniibacillus*, *Symbiobacterium*, *norank_f_Bacillaceae* and *unclassified_f_Paenibacillaceae* were positively related to the GI. Moreover, *Geobacillus* and *Ammoniibacillus* were found at high abundances in bean dregs after thermophilic fermentation and were predicted to be the dominant genera responsible for thermophilic fermentation. Therefore, enhancing the populations of *Geobacillus* and *Ammoniibacillus* could improve the fermentation efficiency and accelerate the maturation of bean dregs.

Additionally, *norank_o_Chloroplast*, *Weissella*, *Leuconostoc* and *Lactococcus* were negatively correlated with the GI. These microorganisms were not conducive to organic fertilizer maturation, and the main reason for this finding was their inability to grow at high temperature.

Ammonia nitrogen was only slightly related to the GI, whereas nitrite nitrogen was positively correlated with the GI. However, the formation of nitrate nitrogen required ammonia nitrogen as a precursor. *Bacillus* and *norank_c_Bacilli* played an important role in the formation of ammonia nitrogen, and *Geobacillus* and *Ammoniibacillus* were positively correlated with nitrite nitrogen, which could promote the transformation of ammonia nitrogen to nitrite nitrogen.

The types of OM could be ranked based on the degree of their positive correlation with the GI as fat > fibre > protein. This finding indicated that changes in the protein content had only a slight effect on the maturity of bean dregs, which might be due to the production of various enzymes during microbial metabolism. However, fat and fibre degradation could enhance the GI and improve the maturation of organic fertilizer. *Geobacillus* and *Ammoniibacillus* were positively correlated with fat loss, whereas *Thermobacillus* can promote fibre loss due to degradation.

These findings suggested that *Geobacillus* and *Ammoniibacillus* improved the maturation of organic fertilizer by promoting fat degradation and nitrite nitrogen formation, and this study provides theoretical guidance for the management of bean dregs during thermophilic fermentation.

## Conclusions

This study screened a thermophilic bacterial strain of *G.toebii* GT-02 at 70 °C for bean dregs thermophilic fermentation. According to the change of the GI, adding GT-02 could shortened mature time of bean dregs from 5 to 3 days by thermophilic fermentation and reduce the loss of total nitrogen. GT-02 also enhanced the conversion of organic nitrogen to ammonia nitrogen and improved the content of nitrate nitrogen. The addition of GT-02 promoted the degradation of fats, proteins, fibres and other organic matters, as well as the generation of humus during fermentation process. The results of microbial diversity showed that the proportion of thermophilic bacteria of *Ammonibacillus* and GT-02 increased at the end of the fermentation process, and it was further found that these thermophilic bacteria were positively correlated with GI value as the organic fertilizer maturity indicator. Therefore, the addition of GT-02 was one of the key factors accelerating the transformation of composition during bean dregs thermophilic fermentation. It was suggested that GT-02 may play an important role in the thermophilic fermentation of fibre-rich materials.

## Data Availability

The datasets used and/or analysed during the current study are available from the corresponding author on reasonable request.

## References

[1] Yang Y (2020). Exploring the microbial mechanisms of organic matter transformation during pig manure composting amended with bean dregs and biochar. Bioresour. Technol..

[2] Zhang L, Sun X (2018). Effects of bean dregs and crab shell powder additives on the composting of green waste. Bioresour. Technol..

[3] Yang Y (2020). Effect of bean dregs amendment on the organic matter degradation, humification, maturity and stability of pig manure composting. Sci. Total Environ..

[4] Wang B (2019). Fabrication of bean dreg-derived carbon with high adsorption for methylene blue: Effect of hydrothermal pretreatment and pyrolysis process. Bioresour. Technol..

[5] Adeyemo SM, Onilude AA (2013). Enzymatic reduction of anti-nutritional factors in fermenting soybeans by *Lactobacillus plantarum* isolates from fermenting cereals. Niger. Food J..

[6] Ying Z, Jianchun H, Huanyu Z, Hui XU, Longfu XU (2013). Research of further processing and comprehensive utilization of soybean dregs. Soybeanence.

[7] Yang Y, Awasthi MK, Ren X, Guo H, Lv J (2019). Effect of bean dregs on nitrogen transformation and bacterial dynamics during pig manure composting. Bioresour. Technol..

[8] Liang C, Das KC, McClendon RW (2003). The influence of temperature and moisture contents regimes on the aerobic microbial activity of a biosolids composting blend. Bioresour. Technol..

[9] Oshima T, Moriya T (2008). A preliminary analysis of microbial and biochemical properties of high-temperature compost. Ann. N. Y. Acad. Sci..

[10] Du G (2021). Exogenous enzyme amendment accelerates maturity and changes microflora succession in horse and wildlife animal manure co-composting. Environ. Sci. Pollut. Res..

[11] Yu Z (2019). Hyperthermophilic composting accelerates the humification process of sewage sludge: Molecular characterization of dissolved organic matter using EEM-PARAFAC and two-dimensional correlation spectroscopy. Bioresour. Technol..

[12] Chang R (2019). Effect of initial material bulk density and easily-degraded organic matter content on temperature changes during composting of cucumber stalk. J. Environ. Sci. (China).

[13] Sarkar S (2010). Effectiveness of inoculation with isolated *Geobacillus* strains in the thermophilic stage of vegetable waste composting. Biores. Technol..

[14] Fang Y (2019). Effect of thermotolerant bacterial inoculation on the microbial community during sludge composting. Can. J. Microbiol..

[15] Poli A (2006). *Geobacillus toebii* subsp. *decanicus* subsp. nov., a hydrocarbon-degrading, heavy metal resistant bacterium from hot compost. J. Gen. Appl. Microbiol..

[16] Rhee SK (2002). Characterization of *Symbiobacterium toebii*, an obligate commensal thermophile isolated from compost. Extremophiles.

[17] Thebti W, Riahi Y, Gharsalli R, Belhadj O (2016). Screening and characterization of thermo-active enzymes of biotechnological interest produced by thermophilic *Bacillus* isolated from hot springs in Tunisia. Acta Biochim. Pol..

[18] Moriya T (2011). *Calditerricola satsumensis* gen. nov., sp. nov. and *Calditerricola yamamurae* sp. nov., extreme thermophiles isolated from a high-temperature compost. Int. J. Syst. Evol. Microbiol..

[19] Cao L (2021). Metagenomic analysis revealed the microbiota and metabolic function during co-composting of food waste and residual sludge for nitrogen and phosphorus transformation. Sci. Total Environ..

[20] Li Y (2020). Odor emission and microbial community succession during biogas residue composting covered with a molecular membrane. Bioresour. Technol..

[21] Yu Z (2018). The distinctive microbial community improves composting efficiency in a full-scale hyperthermophilic composting plant. Bioresour. Technol..

[22] Chen SS (2018). Microbial responses and metabolic pathways reveal the recovery mechanism of an anaerobic digestion system subjected to progressive inhibition by ammonia. Chem. Eng. J..

[23] ISO. *Animal feeding stuffs–Determination of crude fibre content–Method with intermediate filtration.* (2000).

[24] ISO. *Soil Quality—Determination of Nitrate, Nitrite and Ammonium in Field-Moist Soils by Extraction with Potassium Chloride Solution–Part 2: Automated Method with Segmented Flow Analysis*. (2005).

[25] ISO. *Oilseeds—determination of oil content.* (2009).

[26] Sung MH (2002). *Geobacillus toebii* sp. nov., a novel thermophilic bacterium isolated from hay compost. Int. J. Syst. Evol. Microbiol..

[27] Nazina TN (2004). *Geobacillus gargensis* sp. nov., a novel thermophile from a hot spring, and the reclassification of *Bacillus vulcani* as *Geobacillus vulcani* comb. nov.. Int. J. Syst. Evolut. Microbiol..

[28] Coorevits, A. *et al.* Taxonomic revision of the genus *Geobacillus*: Emendation of *Geobacillus, G. stearothermophilus, G. jurassicus, G. toebii, G. thermodenitrificans* and *G. thermoglucosidans* (nom. corrig., formerly 'thermoglucosidasius'); transfer of *Bacillus thermantarcticus* to the genus as *G. thermantarcticus* comb. nov.; proposal of *Caldibacillus debilis* gen. nov., comb. nov.; transfer of *G. tepidamans* to *Anoxybacillus* as *A. tepidamans* comb. nov.; and proposal of *Anoxybacillus caldiproteolyticus* sp. nov. *Int. J. Syst. Evol. Microbiol.* **62**, 1470–1485. 10.1099/ijs.0.030346-0 (2012). 10.1099/ijs.0.030346-0 21856988

[29] Liu T (2020). Influence of fine coal gasification slag on greenhouse gases emission and volatile fatty acids during pig manure composting. Bioresour. Technol..

[30] Chan MT, Selvam A, Wong JW (2016). Reducing nitrogen loss and salinity during 'struvite' food waste composting by zeolite amendment. Bioresour. Technol..

[31] Ogunwande GA, Osunade JA, Adekalu KO, Ogunjimi LA (2008). Nitrogen loss in chicken litter compost as affected by carbon to nitrogen ratio and turning frequency. Bioresour. Technol..

[32] Nie E, Gao D, Zheng G (2020). Effects of lactic acid on modulating the ammonia emissions in co-composts of poultry litter with slaughter sludge. Bioresour. Technol..

[33] Dias BO, Silva CA, Higashikawa FS, Roig A, Sanchez-Monedero MA (2010). Use of biochar as bulking agent for the composting of poultry manure: Effect on organic matter degradation and humification. Bioresour. Technol..

[34] Zhang J, Lu F, Shao L, He P (2014). The use of biochar-amended composting to improve the humification and degradation of sewage sludge. Bioresour. Technol..

[35] Hu TJ (2007). Use of potassium dihydrogen phosphate and sawdust as adsorbents of ammoniacal nitrogen in aerobic composting process. J. Hazard Mater..

[36] Jiang T, Schuchardt F, Li GX, Guo R, Luo YM (2013). Gaseous emission during the composting of pig feces from Chinese Ganqinfen system. Chemosphere.

[37] Gilch S, Meyer O, Schmidt I (2009). A soluble form of ammonia monooxygenase in *Nitrosomonas europaea*. Biometals.

[38] Wehrfritz J-M, Carter JP, Spiro S, Richardson DJ (1996). Hydroxylamine oxidation in heterotrophic nitrate-reducing soil bacteria and purification of a hydroxylamine-cytochromec oxidoreductase from a *Pseudomonas* species. Arch. Microbiol..

[39] Chen W, Westerhoff P, Leenheer JA, Booksh K (2003). Fluorescence excitation-emission matrix regional integration to quantify spectra for dissolved organic matter. Environ. Sci. Technol..

[40] Jouraiphy A, Amir S, El Gharous M, Revel JC, Hafidi M (2005). Chemical and spectroscopic analysis of organic matter transformation during composting of sewage sludge and green plant waste. Int. Biodeter. Biodegr..

[41] Hagemann N (2018). Effect of biochar amendment on compost organic matter composition following aerobic composting of manure. Sci. Total Environ..

[42] Huang M (2018). Investigating binding characteristics of cadmium and copper to DOM derived from compost and rice straw using EEM-PARAFAC combined with two-dimensional FTIR correlation analyses. J. Hazard Mater..

[43] He XS (2014). Insight into the evolution, redox, and metal binding properties of dissolved organic matter from municipal solid wastes using two-dimensional correlation spectroscopy. Chemosphere.

[44] Lv B, Xing M, Yang J, Qi W, Lu Y (2013). Chemical and spectroscopic characterization of water extractable organic matter during vermicomposting of cattle dung. Bioresour. Technol..

[45] Malik SN, Ghosh PC, Vaidya AN, Mudliar SN (2018). Ozone pretreatment of biomethanated distillery wastewater in a semi batch reactor: Mapping pretreatment efficiency in terms of COD, color, toxicity and biohydrogen generation. Biofuels.

[46] Wu J (2017). Identifying the key factors that affect the formation of humic substance during different materials composting. Bioresour. Technol..

[47] Zhang C (2020). Material conversion, microbial community composition and metabolic functional succession during green soybean hull composting. Bioresour. Technol..

[48] Sakai M, Deguchi D, Hosoda A, Kawauchi T, Ikenaga M (2015). *Ammoniibacillus agariperforans* gen. nov., sp. nov., a thermophilic, agar-degrading bacterium isolated from compost. Int. J. Syst. Evol. Microbiol..

[49] Touzel JP, O'Donohue M, Debeire P, Samain E, Breton C (2000). *Thermobacillus xylanilyticus* gen. nov., sp. nov., a new aerobic thermophilic xylan-degrading bacterium isolated from farm soil. Int. J. Syst. Evol. Microbiol..

[50] Munoz-Price LS, Weinstein RA (2008). Acinetobacter infection. N. Engl. J. Med..

